# Gender and Age Interact to Affect Early Outcome after Intracerebral Hemorrhage

**DOI:** 10.1371/journal.pone.0081664

**Published:** 2013-11-27

**Authors:** Odera Umeano, Barbara Phillips-Bute, Claire E. Hailey, Wei Sun, Marisa C. Gray, Briana Roulhac-Wilson, David L. McDonagh, Peter G. Kranz, Daniel T. Laskowitz, Michael L. James

**Affiliations:** 1 School of Medicine, Duke University, Durham, North Carolina, United States of America; 2 Department of Anesthesiology, Duke University, Durham, North Carolina, United States of America; 3 School of Medicine, University of North Carolina – Chapel Hill, Chapel Hill, North Carolina, United States of America; 4 Department of Neurology, Peking University First Hospital, Beijing, PR China; 5 Duke University, Durham, North Carolina, United States of America; 6 Department of Radiology, Duke University, Durham, North Carolina, United States of America; 7 Brain Injury Translational Research Center, Duke University, Department of Neurology, Durham, North Carolina, United States of America; Cardiff University, United Kingdom

## Abstract

**Background:**

Intracerebral hemorrhage (ICH) is a common and devastating form of cerebrovascular disease. In ICH, gender differences in outcomes remain relatively understudied but have been examined in other neurological emergencies. Further, a potential effect of age and gender on outcomes after ICH has not been explored. This study was designed to test the hypothesis that age and gender interact to modify neurological outcomes after ICH.

**Methods:**

Adult patients admitted with spontaneous primary supratentorial ICH from July 2007 through April 2010 were assessed via retrospective analysis of an existing stroke database at Duke University. Univariate analysis of collected variables was used to compare gender and outcome. Unfavorable outcome was defined as discharge to hospice or death. Using multivariate regression, the combined effect of age and gender on outcome after ICH was analyzed.

**Results:**

In this study population, women were younger (61.1+14.5 versus 65.8+17.3 years, p=0.03) and more likely to have a history of substance abuse (35% versus 8.9%, p<0.0001) compared to men. Multivariable models demonstrated that advancing age had a greater effect on predicting discharge outcome in women compared to men (p=0.02). For younger patients, female sex was protective; however, at ages greater than 60 years, female sex was a risk factor for discharge to hospice or death.

**Conclusion:**

While independently associated with discharge to hospice or death after ICH, the interaction effect between gender and age demonstrated significantly stronger correlation with early outcome after ICH in a single center cohort. Prospective study is required to verify these findings.

## Introduction

Intracerebral hemorrhage (ICH) represents up to 10%-15% of strokes in the United States alone, and results in a greater degree of morbidity, mortality, and loss of quality of life than ischemic stroke[1]. However, few improvements in outcome have been realized over the last 20 years, and ICH remains a relatively common and devastating form of cerebrovascular disease. Despite recent priority reports by the National Institutes of Health[2], the American Heart Association[3], and the European Research Network on Intracerebral Hemorrhage[4], ICH continues to be relatively understudied, compared to ischemic stroke, and without any proven therapeutic intervention, as evidenced by several disappointing, yet costly, multicenter clinical trials[5,6]. Similar to ischemic stroke[7], the difficulties of preclinical translation may be partially due to an evolving understanding of the role of sex in neurological recovery and the potential for differential responses of men and women to therapeutics.

Gender differences in outcome have been demonstrated in a variety of acute brain injuries[8-10], but have not been fully characterized in the setting of ICH. Findings from retrospective studies using different European stroke registries[11,12] suggest lower mortality in women after ICH compared to their male counterparts. However, no study to date has directly addressed gender differences in outcome after ICH while controlling for covariates. Further, mechanisms underlying sex-specific outcomes remain unclear, despite ongoing preclinical work demonstrating the neuroprotective effects of female gonadal steroids[13]. Such work has led to clinical trials using exogenous gonadal steroids in both ischemic stroke[14] and traumatic brain injury[15]. 

While early evidence points to gender-specific outcomes after ICH, available data suggest that age affects sex dimorphism in the brain[16-18]. This combined effect of age and gender has not been examined in ICH. Previously, the Duke University Hospital (DUH) Neuroscience Intensive Care Unit (NICU) human database and tissue repository have been used to translate findings to and from the preclinical model of ICH used in the Multidisciplinary Neuroprotection Laboratories at Duke University[19-21]. Thus, this study was proposed to validate preclinical findings of sex dimorphism[13] by using this human database to test the hypothesis that age and gender interact to modify neurological outcomes after ICH. 

## Materials and Methods

### Ethics Statement

Using an existing retrospective database of de-identified patients with ICH admitted to the DUH NICU, analysis was performed after approval by the Duke University Institutional Review Board. Need for written consent by patients for database storage and use of their information for research was waived by the Duke University Institutional Review Board and, therefore, not obtained.

### Study Population

Consecutive patients admitted with computed tomography (CT)-confirmed spontaneous primary ICH from January 2007 through April 2010 were included. Other causes of intracranial hemorrhage were excluded, e.g., hemorrhages secondary to trauma, vascular malformations, coagulopathy, aneurysmal rupture, and tumors. 

Baseline characteristics including demographics, blood pressure, medical co-morbidities, and substance abuse were obtained. Injury severity was assessed on admission based on the initial National Institutes of Health Stroke Scale score (NIHSS)[22], Glasgow Coma Score (GCS)[23], Acute Physiology and Chronic Health Evaluation (APACHE) II score[24], ICH Score[25], and Charlson Index[26]. All data collection was completed by a single individual (WS) and verified by a second (MCG).

Blood pressure was recorded as the initial documented systolic blood pressure on presentation to DUH. Documented comorbidities were gathered from the admitting history and physical in the computerized medical record. Substance abuse was documented from the admitting history and physical or presence of illicit drugs in the admitting urine drug screen. 

Outcome was dichotomized as “favorable” or “unfavorable.” Favorable outcome was defined as discharge to home with or without home health care, discharge to acute rehabilitation center, or discharge to skilled nursing facility. Unfavorable outcome was defined as discharge to hospice or death. 

### Hemorrhage Volumes

For each patient, Digital Imaging and Communications in Medicine (DICOM) format images from the initial head CT were anonymized and loaded in image analysis software (OsiriX version 4.1, Pixmeo, Geneva, Switzerland). A trained image analyst reviewed the images for technical adequacy, and performed manual segmentation of the hematoma volumes (CEH). In cases of intraventricular extension of hemorrhage, the portion of the hematoma within the ventricular system was excluded from the segmented volume. Segmented images were then individually reviewed and edited by a single, blinded neuroradiologist for accuracy (PGK). Automated reports of hematoma volumes were generated by the software and used for analysis.

### Statistical methods

All data were tested for normal distribution. Descriptive statistics are presented as means with standard deviations for continuous variables and as percentages for categorical variables for normally distributed data. Patient characteristics are compared between men and women with *t*-tests for continuous variables, and chi-square tests for categorical variables. Variables missing less than 15% of data were imputed, while retaining the standard deviation and mean. 

Covariates were selected after testing significance in univariate analyses. A multivariable model was constructed to examine the age-by-gender interaction, adjusting for hemorrhage volume, GCS, and history of substance abuse. Odds ratios for the effect of female sex were calculated at selected ages as examples to demonstrate the change in effect with increasing age. In addition, men and women were modeled separately in multivariable analyses, and the effect of age on outcome was assessed within each subgroup. All analyses were performed using SAS v9.2 (SAS, Cary, NC, USA). P < 0.05 was considered significant. As a secondary analysis, NIHSS was included in the multivariable model along with the previously mentioned covariates. Due to missing values, NIHSS was imputed for 67 subjects (preserving the overall mean and standard deviation among the sample). 

## Results

From January 2007 through April 2010, 209 subjects were admitted to DUH NICU with a primary, supratentorial ICH. All subjects had disposition and mortality data at hospital discharge, and were therefore included in the analyses. Demographics of the study population are summarized in Table 1. Initial systolic blood pressure (n = 27), GCS (n = 52), NIHSS (n = 67), ICH Score (n = 110), and APACHE II (n = 107) were missing for some subjects. Men and women in the study population were similar. Although women were younger and more likely to have a history of substance abuse, the percentage of subjects aged 50 years (perimenopausal age for women) and older was similar in both genders.

**Table 1:** Study Population.

	Men (n=97)	Women (n=112)	P value*
Age (years)	65.87 (17.29)	61.11 (14.46)	0.03*
Age > 50 (%)	74.11	75.26	0.85
Caucasian (%)	50.00	50.52	0.94
History of coronary artery disease (%)	15.32	15.46	0.97
History of diabetes (%)	25.23	28.87	0.55
History of hypertension (%)	77.48	80.41	0.61
History of substance abuse (%)	8.93	35.05	<0.0001*
Initial systolic blood pressure (mm Hg)	173.26 (38.69)	183.89 (39.40)	0.08
Initial haemorrhage volume (mL)	37.70 (37.20)	31.98 (37.11)	0.28
Initial Glasgow Coma Score	10.45 (4.33)	10.15 (4.30)	0.07
Initial National Institutes of Health Stroke Scale	12.44 (8.89)	12.61 (9.64)	0.92
Initial Intracerebral Hemorrhage Score	1.89 (1.38)	1.45 (0.94)	0.06
Initial Acute Physiology and Chronic Health Evaluation II Score	15.02 (7.17)	14.78 (7.09)	0.87
Initial Charlson Index	4.11 (2.31)	3.49 (2.49)	0.07
Hospital length of stay (days)	11.86 (13.43)	14.09 (16.21)	0.30
Intensive care unit length of stay (days)	5.80 (6.58)	7.71 (8.67)	0.09
Death by discharge (%)	28.57	23.71	0.43
Death or hospice by discharge (%)	30.36	28.87	0.81

Note: *P values < 0.05 are considered significant. Note: mm = millimeters, Hg = mercury, mL = milliliters, n = number.

In this cohort, 70% of subjects experienced favorable outcome (Table 2) at the time of hospital discharge. Subjects with unfavorable outcome were older, had lower initial GCS, and had higher APACHE II scores, Charlson Index, and NIHSS. Finally, subjects with unfavorable outcome had shorter lengths of hospital stay. 

**Table 2:** Univariate associations between favorable and unfavorable outcomes after intracerebral hemorrhage.

	Favorable (n=147)	Unfavorable (n=62)	P value*
Age	61.80 (16.54)	68.00 (14.65)	0.01*
Age > 50	71.92	83.87	0.07
Caucasian (%)	49.66	61.29	0.12
Women (%)	46.58	45.16	0.83
History of coronary artery disease (%)	13.10	20.97	0.15
History of diabetes (%)	24.83	32.26	0.27
History of hypertension (%)	79.31	77.42	0.76
History of substance abuse (%)	22.54	17.74	0.44
Initial systolic blood pressure (mm Hg)	176.80 (38.12)	184.71 (39.37)	0.22
Initial haemorrhage volume (mL)	33.33 (36.49)	43.01 (42.54)	0.12
Initial Glasgow Coma Score	12.04 (3.75)	7.13 (3.44)	<0.0001*
Initial National Institutes of Health Stroke Scale	9.70 (7.60)	20.88 (8.55)	<0.0001*
Initial Intracerebral Hemorrhage Score	1.63 (1.17)	1.90 (1.37)	0.32
Initial Acute Physiology and Chronic Health Evaluation II Score	12.94 (6.33)	18.20 (7.00)	0.0001*
Initial Charlson Index	3.45 (2.38)	4.71 (2.29)	0.0005*
Intensive care unit length of stay (days)	6.92 (8.08)	5.92 (6.05)	0.33
Hospital length of stay (days)	15.02 (15.82)	7.21 (8.91)	<0.0001*

* P values < 0.05 were considered significant. Note: Hg = mercury, mL = milliliters, mm = millimeters, n = number.

**Figure 1:**
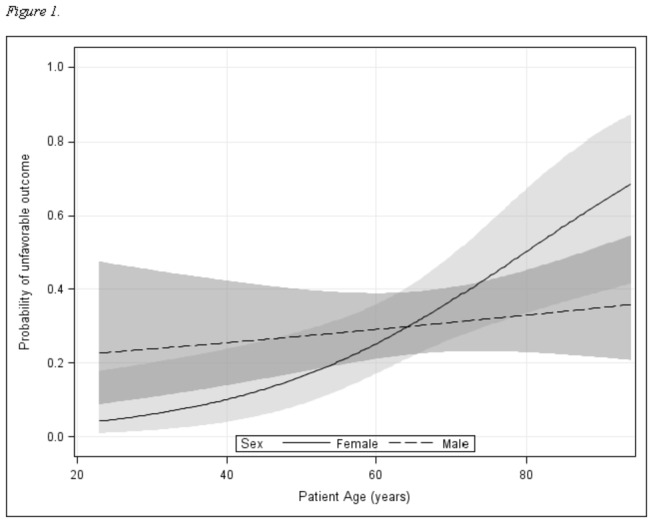
Predicted probability of an unfavorable outcome for men and women. Probabilities are derived from a multivariable model including an age-by-gender interaction. Shaded areas represent 95% confidence intervals. The significant interaction term indicates that the association between gender and unfavorable outcome depends on age. For younger patients, female sex is protective; at older ages, female sex is a risk factor.

To examine the relationship between gender, age, and outcome, a multivariable model was created (Table 3). Covariates included history of substance abuse, hemorrhage volume, and GCS. In this multivariable model, GCS had a negative association with unfavorable outcome after ICH. While age was associated with unfavorable outcome in this model, gender was not. However, a significant age-by-gender interaction was found when predicting unfavorable outcome after ICH, indicating that the effect of gender on outcome differs by age, and vice versa. Thus, for each point along the age curve, the odds ratio for gender was different. For example, women at the age of 30 years (premenopausal) had an odds ratio for an unfavorable outcome of 0.09; at the age of 50 years (perimenopausal), the odds ratio was 0.44; and at the age of 70 years (postmenopausal), the odds ratio for women was 2.25. Notably, the confidence intervals for each of these odds ratios includes 1, indicating that the risk for unfavorable outcome was not significantly different in men versus women at any of these ages. However, the significant interaction term between age and gender indicates that the slopes of the lines vary between men and women (Figure 1); thus, age affects risk of unfavorable outcome differently in men than in women with women experiencing a greater increase in risk as they age. 

**Table 3:** Multivariate model for predicting unfavorable outcome.

	Odds Ratio		95% Confidence Limits			P-value	
History of substance abuse	1.34		0.37		4.84	0.66	
Hemorrhage volume (mL)	1.01		1.00		1.02	0.13	
Glasgow Coma Score	0.72		0.64		0.81	<0.0001*	
Gender						0.04*	
Age						0.12	
Age-by-gender interaction	0.09		0.01		1.27	0.002*	
Female gender at age 30 years							
Female gender at age 50 years	0.44		0.10		1.97		
Female gender at age 70 years	2.25		0.81		6.24		

The odds ratio for age-by-gender interaction differs for each point along the age curve, e.g., 30 (premenopausal), 50 (menopausal), and 70 (postmenopausal). C-index for the multivariable model is 0.88. *P values < 0.05 were considered significant. Note: mL = milliliter.

To further investigate this interaction, men and women were modelled separately in multivariable models using the same covariates. For women, the effect of age was significant (p = 0.0003, OR 1.13, 95% CI 1.058-1.21), while for men, the effect of age was non-significant (p = 0.27, OR 1.02, 95% CI 0.98-1.07). This confirms the multivariable analysis finding that the risk for unfavorable outcome increased significantly in women with increasing age, but not in men. 

Finally, after imputing the missing NIHSS values for 67 study subjects, the age-by-gender interaction effect remained significant (p = 0.02). GCS (p < 0.01), age (p = 0.01) and gender (p = 0.04) also retained significance in this multivariable model. 

## Discussion

These findings support the hypothesis that age and gender interact to modify outcome after ICH. Although factors that influence recovery from ICH have been explored in major clinical trials[5,6], the potential interaction of gender and age has been largely overlooked. Although sex differences in the brain’s response to multiple acute brain injury types have been demonstrated[8-10,27], these differences are not well characterized in ICH. 

In epidemiologic studies, patients with ICH have most often been treated as a subset of larger cohorts of ischemic stroke patients[1,11,27-29]; thus, confirmation of gender differences in ICH is difficult. However, sex dimorphism in recovery from brain injury is biologically plausible. Female gonadal steroids have been shown to be neuroprotective in a variety of preclinical models[30,31], including ICH[32-34]. Thus, signs point to differences in ICH, but mechanistic data are limited. The present findings are the first to indicate that sex-specific effects may be related to the age at which outcome is assessed. 

While knowledge about the effects of gender continues to emerge, the effect of aging in ICH has received substantial attention. Incidence of ICH varies by age[35]; and amyloid angiopathy, as a cause of ICH, is directly related to aging[36]. Further, age is strongly associated with outcome after ICH[25]. This association may be related to aging effects on hematoma volume[37] and neuroinflammation[38,39].

Most of the subjects in the present study experienced a favorable outcome. Short-term outcome measures and exclusion of patients with infratentorial hemorrhage may explain this finding. Patients who experienced a favorable outcome had higher GCS scores, lower APACHE II scores, lower NIHSS, and longer hospital stays. Although GCS and NIHSS are more traditional metrics, APACHE II scores have been associated with outcomes in critical neurological illness[40]. Differences in hospital length of stay may be explained by higher mortality at an earlier time point in the cohort with an unfavorable outcome, i.e., 50% of subjects with unfavorable outcome died in less than 72 hours from ICH onset. 

Importantly, gender-specific differences in age of ICH onset were observed in the present study population. However, while women in the present study population were moderately younger, no distinction was made for prior history of ICH. Prior epidemiologic studies have found no gender difference in lifetime risk of ICH, although some have found that men are more likely to suffer initial ICH at an earlier age[27]. While the present study focused on early outcome, future study should address potential gender differences in both recurrence rates and ICH etiology since recurrence can vary dramatically based on etiology[41]. Regardless, the percentage of subjects over the age of 50 years (the approximate median age of menopause) was not different between the genders. Further, men and women did not differ in the rate of comorbidities, ICH severity, or ICH volume.

In addition, women were more likely to have a history of substance abuse in the present study. Use of cocaine and amphetamines is a known risk factor for developing ICH[42], and there are gender differences in overall rates of substance abuse[43]. However, gender differences in substance abuse among patients with ICH have not been previously evaluated. This study was not designed to explore this question; thus, specific drugs of abuse, e.g., cocaine and alcohol, were not individually analyzed. However, history of substance abuse did not significantly affect the predictive model and may not play a crucial role in determining outcome after ICH. Despite this, gender-specific substance abuse patterns in patients with ICH should be more fully explored in future studies.

GCS was used to gauge ICH severity in the multivariate model. Although Charlson Index, APACHE II, and NIHSS were also significantly correlated with outcome, these measures were not performed on all subjects at presentation. Though GCS is a reliable indicator of the severity of neurological insult[44], the combination of NIHSS and GCS can be strongly predictive of mortality after ICH[45]. Thus, in a secondary analysis, the incorporation of NIHSS was assessed using imputed values for missing data. For both multivariate models, i.e., with and without imputed NIHSS data, the results were consistent. Finally, while one accepted measure of stroke severity is the NIHSS, the Charlson Index and APACHE II scores may be useful in determining the overall degree of critical illness in a given patient. Although the number of variables for inclusion in the primary regression model was limited by the number of subjects with unfavorable outcome, secondary analyses were performed including APACHE II or Charlson Index as replacements for GCS. Results from these analyses were consistent with the primary analysis.

In addition to the limitations inherent to retrospective analyses, the implications of underlying pathophysiology of ICH, e.g., cerebral edema[19,46] and hematoma formation[47], for early outcomes could not be assessed from this dataset. Further, though short-term outcomes provide clues about the trajectory of recovery, long-term outcomes were not captured. Finally, due to sample size, the number of variables analyzed in the model was limited by statistical power. While multiple other factors may be involved in predicting outcome after ICH, complete evaluation would require much larger sampling.

## Conclusions

These data support the hypothesis that gender and age interact to modify early outcome after ICH. While larger, prospective studies are needed to validate these findings, this study represents a first step toward mechanistic investigations to lay the foundation for the development of sex-tailored therapies. 
